# Signaling Pathways Mediating Bradykinin-Induced Contraction in Murine and Human Detrusor Muscle

**DOI:** 10.3389/fmed.2021.745638

**Published:** 2022-01-20

**Authors:** Kinga Borsodi, Helga Balla, Péter József Molnár, Ádám Lénárt, István Kenessey, András Horváth, Attila Keszthelyi, Miklós Romics, Attila Majoros, Péter Nyirády, Stefan Offermanns, Zoltán Benyó

**Affiliations:** ^1^Institute of Translational Medicine, Semmelweis University, Budapest, Hungary; ^2^Department of Urology, Semmelweis University, Budapest, Hungary; ^3^2^nd^Department of Pathology, Semmelweis University, Budapest, Hungary; ^4^Department of Pharmacology, Max Planck Institute for Heart and Lung Research, Bad Nauheim, Germany

**Keywords:** urinary bladder smooth muscle, bradykinin, signal transduction, Rho-kinase, detrusor reactivity

## Abstract

Bradykinin (BK) has been proposed to modulate urinary bladder functions and implicated in the pathophysiology of detrusor overactivity. The present study aims to elucidate the signaling pathways of BK-induced detrusor muscle contraction, with the goal of better understanding the molecular regulation of micturition and identifying potential novel therapeutic targets of its disorders. Experiments have been carried out on bladders isolated from wild-type or genetically modified [smooth muscle-specific knockout (KO): Gα_q/11_-KO, Gα_12/13_-KO and constitutive KO: thromboxane prostanoid (TP) receptor-KO, cyclooxygenase-1 (COX-1)-KO] mice and on human bladder samples. Contractions of detrusor strips were measured by myography. Bradykinin induced concentration-dependent contractions in both murine and human bladders, which were independent of secondary release of acetylcholine, ATP, or prostanoid mediators. B_2_ receptor antagonist HOE-140 markedly diminished contractile responses in both species, whereas B_1_ receptor antagonist R-715 did not alter BK's effect. Consistently with these findings, pharmacological stimulation of B_2_ but not B_1_ receptors resembled the effect of BK. Interestingly, both Gα_q/11_- and Gα_12/13_-KO murine bladders showed reduced response to BK, indicating that simultaneous activation of both pathways is required for the contraction. Furthermore, the Rho-kinase (ROCK) inhibitor Y-27632 markedly decreased contractions in both murine and human bladders. Our results indicate that BK evokes contractions in murine and human bladders, acting primarily on B_2_ receptors. Gα_q/11_-coupled and Gα_12/13_-RhoA-ROCK signaling appear to mediate these contractions simultaneously. Inhibition of ROCK enzyme reduces the contractions in both species, identifying this enzyme, together with B_2_ receptor, as potential targets for treating voiding disorders.

## Introduction

Bradykinin (BK) is a nonapeptide pro-inflammatory mediator with a diverse set of functions, including regulation of smooth muscle tone. It has been reported that BK induces smooth muscle contraction in the vascular system, airways (1, 2), ureter (3), uterus (4), colon (5), and prostate (6).

Bradykinin exerts its effects in smooth muscle tissues via two major subtypes of BK receptors: B_1_ and B_2_ (7). Despite their structural similarity, B_1_ and B_2_ receptors differ greatly regarding their expression profiles in tissues and their function as well (8). The amount of B_1_ receptors in healthy human tissues is negligible, however, as a consequence of inflammatory stimuli (e.g., IL-1β), tissue injury, and endogenous factors, their expression may increase rapidly (9). In contrast, B_2_ receptors' presence is constitutive and predominant under physiological conditions (8, 10). Expression of both B_1_ and B_2_ receptors has been reported in urinary bladder smooth muscle (UBSM), however, their role and signaling pathways remain to be elucidated (11). B_1_ and B_2_ receptors are members of the seven-transmembrane-domain, type 1 G protein-coupled receptor (GPCR) family, representing a major class of drug targets (10, 12).

Bradykinin is a multi-faceted mediator of urinary bladder functions as its receptors are expressed in practically all cell types of the bladder, including the mucosa, UBSM, afferent nerve fibers as well as cells of innate and acquired immunity (11–14). Interestingly, however, no voiding abnormalities have been reported in mice lacking both the B_1_ and B_2_ receptor genes (15) or in control rat bladders pretreated with the B_2_ receptor antagonist HOE-140 (16), indicating a limited role of BK in the micturition reflex under physiological conditions. In contrast, BK has been reported to increase bladder activity (17); furthermore, the expression of its receptors is increased markedly in association with voiding disorders (16, 18). Bradykinin has been proposed as a mediator of augmented bladder contractions in a rat model of overactive bladder (OAB) syndrome (19). According to this study, B_1_ and B_2_ receptor agonists induced higher contractile responses in bladder strips of the OAB as compared to the sham-operated group. In addition, under the above-mentioned conditions, non-resident inflammatory cells capable of facilitating BK production and sensitivity to BK may appear in the bladder, further enhancing the possibility of BK-related functional changes.

Although it has been known for a long time that BK is present in the urine, assumingly, it is not the primary source of BK's effect on bladder smooth muscle, as the intact urothelium forms a tight barrier highly impermeable for compounds in the urine. However, the mucosa layer of the bladder wall exhibits diverse functions in regulating bladder tone, including releasing numerous mediator molecules. Saban et al. reported that BK is released from the bladder mucosa under physiological conditions, moreover, the mucosa layer also plays a major role in degrading the peptide (20). Under pathological conditions, the balance of peptide release and degradation may be disturbed, and either overproduction or decreased degradation of the peptide may lead to symptoms of bladder smooth muscle overactivity. In addition, BK may facilitate the release of other inflammatory mediators, like prostanoids, which can induce detrusor muscle contraction (21, 22).

The general principles of smooth muscle contraction apply to the urinary bladder, and in order to understand better the actions of BK we focused on the signaling pathways of UBSM contraction. Generally, smooth muscle contraction is initiated by the elevation of intracellular Ca^2+^ concentration entering the cytoplasm either via cell membrane channels or from the sarcoplasmic reticulum (SR), resulting in the activation of myosin light chain (MLC) kinase that phosphorylates MLC, eventually leading to cross-bridge cycling between actin and myosin. A common stimulus for the elevation of intracellular Ca^2+^ concentration is GPCR activation. Gα_q/11_-coupled receptors stimulate phospholipase C-ß (PLC-ß) activity resulting in formation of inositol triphosphate and diacylglycerol (DAG). Inositol triphosphate binding to its receptors on the SR is the stimulus for intracellular Ca^2+^ release, which in turn may induce the opening of store-operated Ca^2+^ channels of the plasma membrane resulting in Ca^2+^-influx and further activation of MLC kinase.

Myosin light chain phosphorylation is also regulated by MLC phosphatase, which removes the phosphate from MLC and promotes relaxation. Gα_12/13_-coupled receptor signaling involves activation of the small G protein RhoA and consequently Rho-kinase (ROCK), which inactivates MLC phosphatase leading to a sustained contraction (23). This pathway is often referred to as the Ca^2+^-sensitizing pathway, as an increase in intracellular Ca^2+^ concentration is not needed for the contraction. The ROCK enzyme has gained attention recently in disorders associated with lower urinary tract smooth muscle contractility (24, 25). In addition, ROCK activity may be enhanced under pathological conditions, for instance, in cystitis (26) and during aging (27) highlighting its possible role in bladder dysfunctions.

This paper focuses on signal transduction pathways contributing to BK-induced detrusor smooth muscle contraction and their potential role in regulating bladder tone. Intracellular signaling of BK-induced contraction has been examined in several smooth muscle tissues (2, 4, 12). We aimed to elucidate whether the same pathways mediate its effect in the detrusor muscle as well and to find potential targets within the signaling cascade of BK-evoked contractions for intervention in UBSM dysfunctions.

## Materials and Methods

### Animals

All procedures were carried out according to the guidelines of the Hungarian Law of Animal Protection (28/1998) and were approved by the Government Office of Pest County (Permission number: PEI/001/2709-13/2014). Urinary bladders were obtained from adult (90-120-day-old, 30-35 g) male wild-type (WT) mice (C57BL/6N mice from Charles River Laboratories, Isaszeg, Hungary) and from animals deficient for the cyclooxygenase (COX)-1 enzyme (COX-1-KO) or thromboxane prostanoid (TP) receptor (TP-KO) or from mice with induced smooth muscle-specific deficiency of Gα_q/11_ or Gα_12/13_ proteins as described previously (Gα_q/11_-KO and Gα_12/13_-KO) (28, 29).

Cyclooxygenase-1 enzyme mice were kindly provided by Ingvar Bjarnason (Department of Medicine, Guy's, King's College, St. Thomas' School of Medicine, London, United Kingdom), and prostanoid receptor-deficient mice were from Shuh Narumiya (Kyoto University, Kyoto, Japan). In the case of the COX-1-KO studies, littermate COX-1^+/+^ animals, whereas in TP-KO experiments, WT C57Bl/6N mice served as controls, as the TP-KO strain has been previously backcrossed with C57Bl/6N mice for more than 10 generations.

The mouse lines with smooth muscle-specific inducible deletion of the Gα_q/11_ or Gα_12/13_ proteins were generated on Gα_11_-deficient (G$\alpha_{11}^{-/-}$) or Gα_12_-deficient (G$\alpha_{12}^{-/-}$) background (30, 31) with floxed alleles of the genes coding Gα_q_ (Gnaq^flox/flox^) or Gα13 (Gnα13^flox/flox^), and expressing a fusion protein of the Cre-recombinase with a modified estrogen receptor-binding domain (Cre-ERT2) (32) under the control of the smooth muscle myosin heavy chain (SMMHC) promoter. Deletion of Gnaq or Gna13 was induced by intraperitoneal tamoxifen treatment (1 mg/day for five consecutive days) in SMMHC-CreERT2^+/−^; Gnaq^flox/flox^; Gna11^−/−^ and SMMHC-CreERT2^+/−^; Gna12^−/−^; Gna13^flox/flox^ mice, respectively, as described previously elsewhere (28, 29). Mice with floxed alleles but without Cre expression served as controls. More precisely, tamoxifen-treated SMMHC-CreERT2^−/−^; Gnaq^flox/flox^; Gna11^+/+^ and SMMHC-CreERT2^−/−^; Gna12^+/+^; Gna13^flox/flox^ mice served as controls and are referred to as Gα_q/11_-CTRL and Gα_12/13_-CTRL.

### Human Tissues

All procedures involving human urinary bladder tissues have been approved by the Scientific and Research Committee of the Medical Research Council of Hungary (License No.: 21545-2/2019/EKU). Human urinary bladder tissues were obtained from 19 patients (15 males, 4 females; mean age of 65.5 ± 9.3 years, range between 44 and 78 years) undergoing open radical cystectomy due to muscle-invasive bladder malignancy after having obtained written patient consent. None of the patients had any urodynamic disorders, symptoms of OAB syndrome, or was taking drugs for OAB.

Following the surgical removal of the bladders, they were immediately placed in physiological saline solution and transported to the 2^nd^ Department of Pathology of the Semmelweis University, Budapest. Here, the healthy, tumor-free whole bladder wall tissue was provided by uro-pathologists within approximately 15–20 min following removal of the bladders from patients as described previously (33). The healthy bladder tissue was immediately placed into room temperature Hank's Balanced Salt Solution (HBSS) and transported to our myograph laboratory, where preparation of the smooth muscle strips was performed without delay. Overall, myographic experiments started within 45–60 min following bladder removal from the patients.

### Preparation of Smooth Muscle Strips

Mice were euthanized by cervical dislocation under general anesthesia [i.p., Ketamine (300 mg/kg) + Xylazine (30 mg/kg)], the urinary bladders were removed from a lower midline incision and were placed into Krebs solution (119 mM NaCl, 4.7 mM KCl, 1.2 mM KH_2_PO_4_, 2.5 mM CaCl_2_·H_2_O, 1.2 mM MgSO_4_·7H_2_O, 20 mM NaHCO_3_, 0.03 mM EDTA, and 10 mM glucose, pH 7.4) at room temperature. Under a dissection microscope (M3Z; Wild Heerbrugg AG, Gais, Switzerland), adipose and connective tissues were removed from the serosal surface. Bladders were cut into four strips of equal lengths and, the mucosa layer was also gently removed to prevent the potential release of paracrine factors from the mucosal epithelium or submucosa and avoid tension changes related to myofibroblasts.

Human urinary bladder specimens were also placed into Krebs solution (same as described before, at room temperature) during the preparation. Under a dissection microscope, the serosal tissue and the mucosal layer were removed. The isolated detrusor muscle specimens were cut into equal, approximately 3 × 2 × 1 mm strips for myography.

### Measurement of Bladder Contractility

Both murine and human detrusor muscle strips were mounted on two parallel, horizontal stainless-steel tissue-holding needles of a myograph (needle diameter 200 μm, 610 M Multi Wire Myograph System, Danish Myo Technology A/S, Aarhus, Denmark). Chambers were filled with 6 ml of Krebs solution aerated with carbogen (mixture of 5% CO_2_ and 95% O_2_) at 37°C. Detrusor muscle contractions were recorded under isometric conditions. Every experiment started with a 60-min resting period while the strips were stretched to and stabilized at a passive tension of 5 mN (murine) or 3 mN (human). After the resting period, UBSMs were challenged twice with 124 mM K^+^-containing Krebs solution to examine the viability of the tissues. The contractile effect of 124 mM K^+^ was comparable in the detrusor strips obtained from the WT and the genetically modified mouse lines (Supplementary Figure 1 at https://doi.org/10.6084/m9.figshare.16815061.v5). After several washes with normal Krebs solution, the contractile effects of BK (10^−10^-10^−4^ M), Lys-[Des-Arg^9^]-bradykinin (B_1_ receptor agonist, 10^−5^ M), [Phe^8^Ψ(CH-NH)-Arg^9^]-bradykinin (B_2_ receptor agonist, 10^−5^ M), carbamoylcholine chloride [carbachol (CCh, 10^−6^ M)], α,β-methyleneadenosine 5′-triphosphate [α,β-meATP, ATP-analog (10^−5^ M)] was measured. Some of the strips were preincubated with one of the following inhibitors without washing out: R-715 (Ac-Lys-Arg-Pro-Pro-Gly-Phe-Ser-DβNal-Ile, B_1_ receptor antagonist, 10^−6^ M, 20 min), HOE-140 (icatibant) (D-Arg-Arg-Pro-Hyp-Gly-Thi-Ser-D-Tic-Oic-Arg, B_2_ receptor antagonist, 10^−6^ M, 20 min), atropine [muscarinic-acetylcholine (ACh)-receptor antagonist, 10^−6^ M, 20 min], pyridoxalphosphate-6-azophenyl-2′,4′-disulfonate (PPADS; P2 purinergic receptor antagonist, 10^−5^ M, 20 min), Y-27632 [ROCK inhibitor, 10^−5^ M, 20 min], indomethacin [non-isoform-selective COX inhibitor, 10^−5^ M, 20 min], *N*-[2-Cyclohexyloxy-4-nitrophenyl]methanesulfonamide [NS-398, COX-2 inhibitor 10^−5^ M, 20 min]. When acetic acid, dimethyl sulfoxide (DMSO), or saline was the solvent of the inhibitor, they were applied in matched concentrations as vehicle control. The final concentration of acetic acid in the tissue bath was 0.1 mM, while that of DMSO was 0.1%. Since repeated use of BK or its analogs in the same specimen is problematic due to the rapid desensitization and the viability of the tissues may decline in a prolonged experiment, we performed unpaired, time-control experiments in this study. Finally, bladder strips were exposed to 124 mM K^+^-containing Krebs solution to retest the viability of the detrusor strips. Agonist-induced tension changes were normalized to the reference contraction induced by 124 mM K^+^-containing Krebs solution (second administration). A schematic diagram of our experimental protocol is demonstrated in Supplementary Figure 2 (at https://doi.org/10.6084/m9.figshare.16815097.v2).

MP100 system and AcqKnowledge 3.9.2 software from Biopac System (Goleta, CA) were used for the acquisition and analysis of myographic measurements. The moving average smoothening function of the software was applied on recordings solely in order to eliminate the noises arising from the bubbling of the medium and to reduce the high frequency—low amplitude spontaneous tension oscillations. The parameters of the smoothening filter were carefully chosen in order to eliminate only the noises but not to alter the amplitude of the BK-induced responses—the baseline and peak values were always compared before and after the smoothing. The sample rate of the recordings was 10 samples/s (10 Hz), the smoothing factor was between 10 and 40 samples. Spontaneous micro-contractions of the bladder strips were observed occasionally, however, they were not reproducible, thus these data were not considered for demonstration or evaluation in the present study.

### Drugs and Solutions

Bradykinin was purchased from Bachem (Bubendorf, Switzerland) and dissolved in acetic acid (0.1 M) to stock solutions of 10^−2^ M. Lys-[Des-Arg^9^]-bradykinin, [Phe^8^Ψ(CH-NH)-Arg^9^]-bradykinin, HOE-140, and R-715 were purchased from Tocris (Bristol, UK) and were dissolved in saline. Stock solutions of Lys-[Des-Arg^9^]-bradykinin, [Phe^8^Ψ(CH-NH)-Arg^9^]-bradykinin, and R-715 were 10^−3^ M, whereas due to its poor solubility in water, stock solutions of HOE-140 were 5 × 10^−4^ M. Carbachol was from Sigma-Aldrich (St. Louis, MO) and dissolved in saline to a stock solution of 2 × 10^−1^ M. Atropine (atropinum sulfuricum) was purchased from Egis Pharmaceutical PLC (Budapest, Hungary) and was diluted in water to a stock solution of 1.44 × 10^−4^ M. α,β-Methyleneadenosine 5′-triphosphate, PPADS, and Y-27632 were purchased from Cayman Chemical (Ann Arbor, MI) and all substances were dissolved in saline (α,β-meATP: 10^−2^ M, PPADS: 10^−2^ M, and Y-27632: 10^−3^ M). Indomethacin was purchased from Sigma-Aldrich (St. Louis, MO) and dissolved in DMSO (10^−2^ M stock concentration), as its aqueous solutions are quite unstable (34). NS-398 was also purchased from Sigma-Aldrich (St. Louis, MO), and DMSO was applied as solvent for preparing a 10^−2^ M stock solution.

### Data Analysis and Statistics

The maximum contraction was defined as the peak value of tension developed after the addition of agonists. Average curves of individual contraction responses were also determined and presented on the left side of the figures, where they were plotted as mean values. All data are presented with the median values except concentration-response curves, in which cases mean ± SEM were used. For mouse concentration-response curve analysis, curves were fitted for data from each experiment, thus E_max_ and EC_50_-values were determined for each curve, and the average values were calculated thereafter. In the case of human concentration-response correlation, curves were fitted on data gained from numerous experiments, as human tissues exhibit more variable responses which made curve-fitting from each individual experiment difficult.

For statistical analysis, data sets were subjected to non-parametric testing, as in the case of small sample sizes and skewed data, parametric testing might not be appropriate. In the case of comparing two data sets the Mann-Whitney test, while in the case of comparing several data sets, the Kruskal-Wallis test was performed for determining the corresponding *p*-values. The following formula was used for demonstrating case numbers: *n* = *x*/*y*, where *x* represents the number of the bladder strips and *y* indicates the number of bladders. Statistical analysis and graph plotting were performed with GraphPad Prism software (v.6.07; GraphPad Software Inc., La Jolla, CA, USA), and *p* < 0.05 was considered a statistically significant difference.

## Results

### Bradykinin Induces Concentration-Dependent Contractions in Mouse and Human Bladders With Similar Characteristics

**Figure 1:**
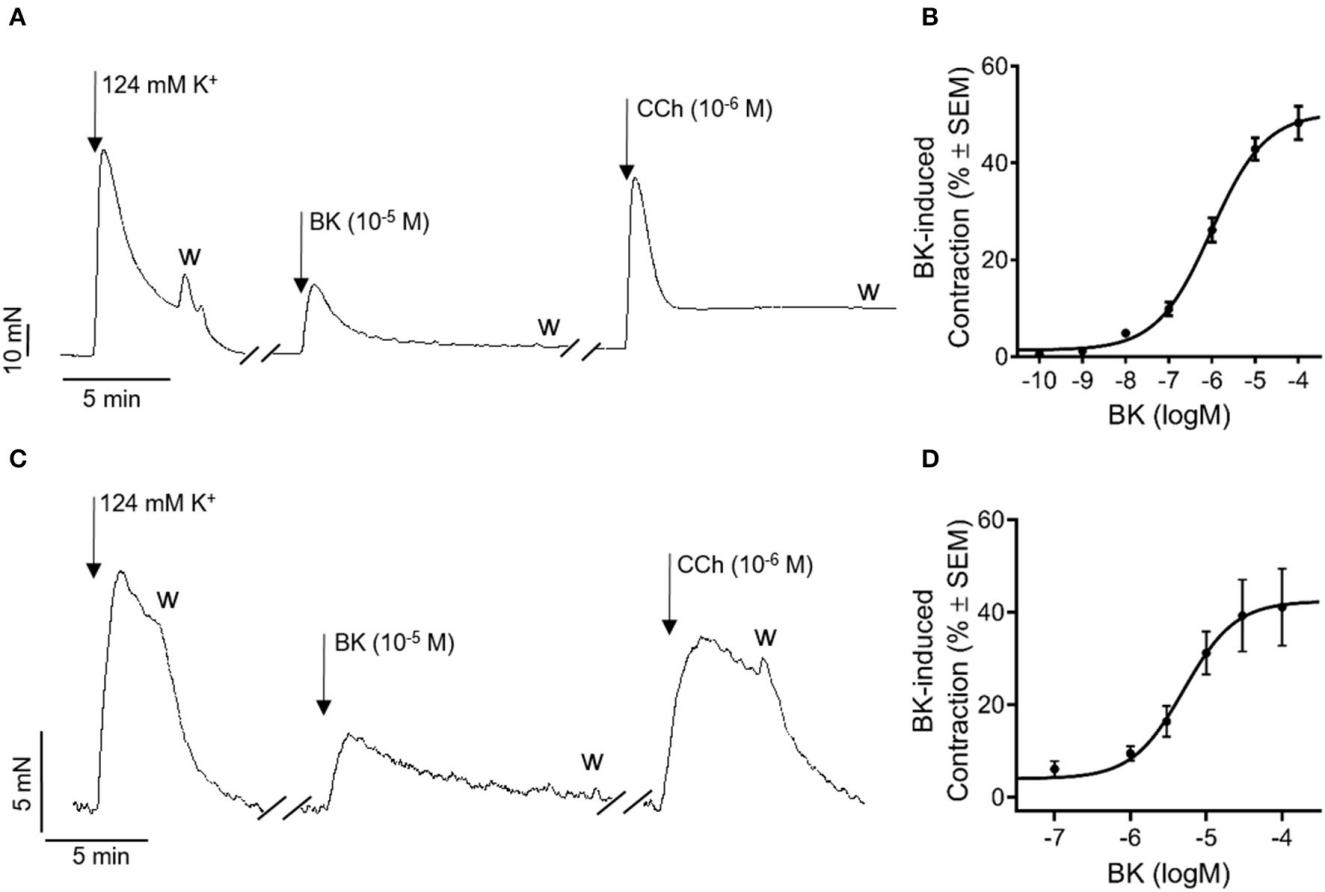
Bradykinin induces concentration-dependent contractions in mouse and human bladders with similar characteristics. Original trace: BK (10^−5^ M) evoked contractions in isolated murine detrusor smooth muscle strips which were comparable to the responses evoked by the muscarinic ACh receptor agonist CCh (10^−6^ M). **(A)** Concentration-response curve of BK in murine urinary bladder strips (E_max_: 52.4% EC_50_: 1.2 μM) **(B)** Case numbers: *n* (10^−10^) = 2/2, *n* (10^−9^) = 3/3, *n* (10^−8^) = 3/3, *n* (10^−7^) = 6/6, *n* (10^−6^) = 9/7, *n* (10^−5^) = 4/4. *n* (10^−4^) = 10/10. Original trace: In accordance with our results gained from murine bladder strips, BK (10^−5^ M) evoked contraction in human detrusor smooth muscle as well. Moreover, the amplitude of the contractile effect was comparable to that induced by the muscarinic-acetylcholine-receptor agonist CCh (10^−6^ M). **(C)** Concentration-response curve representing BK's contractile effect in human detrusor smooth muscle. (E_max_: 42.4%; EC_50_: 5.1 μM), **(D)** Case numbers: *n* (10^−7^) = 7/3, *n* (10^−6^) = 7/3, *n* (3 × 10^−6^) = 11/4, *n* (10^−5^) = 13/6, *n* (3 × 10^−5^) = 15/5, *n* (10^−4^) = 11/4.

First, we aimed to evaluate the effect of BK in murine and human urinary bladders. Bradykinin induced marked, transient contraction in the mouse bladder, although it did not reach the level of the response evoked by CCh, a stable analog of ACh, the main physiological mediator of detrusor muscle contraction (Figure 1A). The effect of BK was dose-dependent, with the EC_50_ of 1.24 μM and E_max_ of 52.4%, expressed as the percentage of the reference contraction induced by 124 mM KCl (Figure 1B). In human bladder strips, BK induced comparable contractions with a similar ratio to the CCh's effect to that observed in mice (Figure 1C). Bradykinin-induced contractions were also dose-dependent in human bladders with an EC_50_ of 5.1 μM and E_max_ at 42.4% (Figure 1D). As repeated administration of BK appeared to desensitize BK receptors both in murine and human bladders, the dose-response curves have been obtained by applying only one single concentration of BK to each muscle strip and also in the further experiments, we avoided repeated administration of BK. Based on the dose-response relationship presented in Figures 1B,D, we decided to apply BK in subsequent experiments in a concentration of 10 μM, which induces a submaximal contractile effect enabling the determination of the signaling pathways involved.

### Signaling Pathways of Bradykinin-Induced Detrusor Muscle Contractions

**Figure 2:**
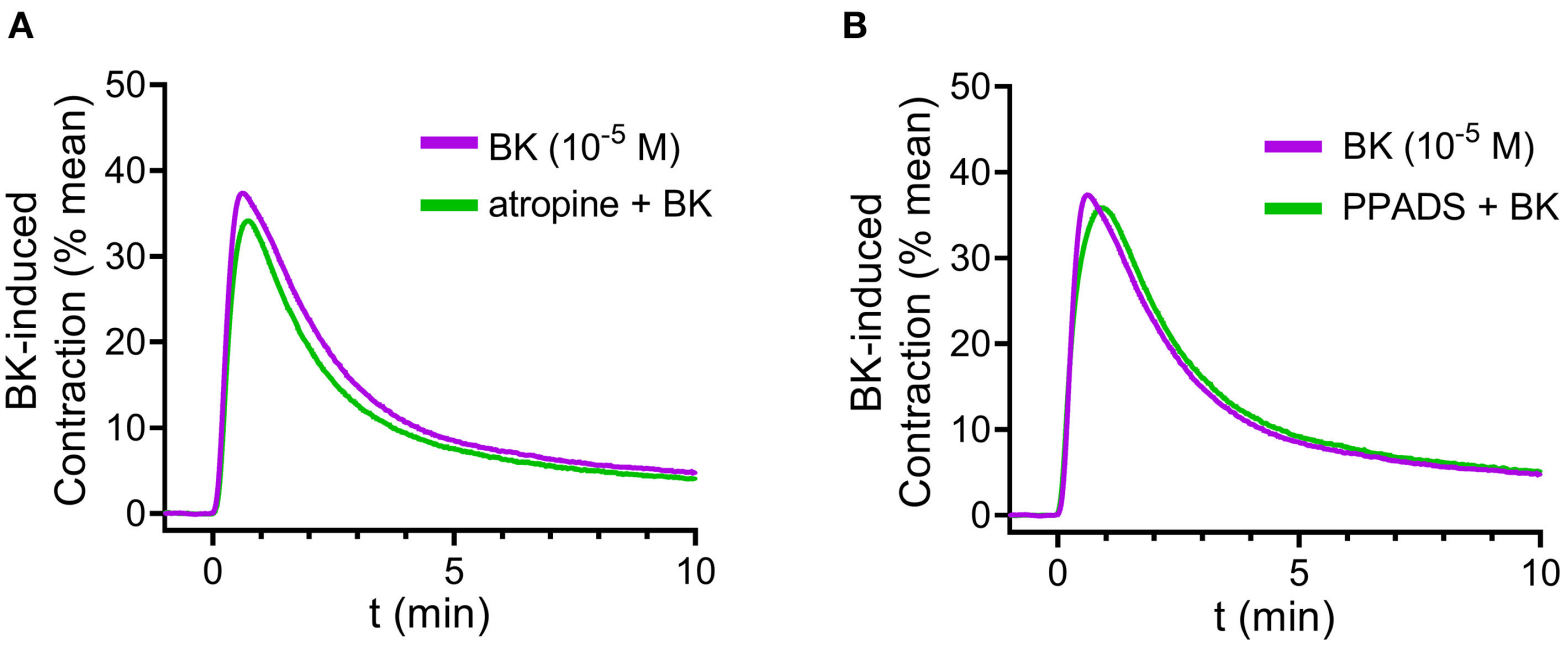
Bradykinin-induced detrusor muscle contraction is independent of purinergic or cholinergic neurotransmission. Neither inhibition of purinergic receptors with PPADS (10^−5^ M, 20 min incubation) nor the muscarinic receptor antagonist atropine (10^−6^ M, 20 min incubation) altered detrusor contraction induced by BK (10^−5^ M). **(A,B)** Case numbers: **(A)**: BK: *n* = 6/6, atropine + BK: *n* = 7/7, **(B)**: BK: *n* = 6/6, PPADS + BK: *n* = 8/8.

First, in order to test whether secondary ACh or ATP release from parasympathetic nerve fibers may mediate the effects of BK, we applied the muscarinic ACh receptor antagonist atropine (10^−6^ M, 20 min incubation) (Figure 2A) or the P2 purinergic receptor antagonist PPADS (10^−5^ M, 20 min) (Figure 2B). Neither atropine nor PPADS altered the effect of BK in mouse detrusor muscle, suggesting that the BK-induced contractile responses are independent of secondary ACh or ATP release (Figures 2A,B). To verify the effectiveness of atropine and PPADS, we applied the specific purinergic agonist α,ß-meATP, and the muscarinergic agonist CCh following preincubation with either PPADS or atropine, respectively (Supplementary Figures 3A,B at https://doi.org/10.6084/m9.figshare.14718657.v4). Pyridoxalphosphate-6-azophenyl-2′,4′-disulfonate abolished the contractile effect of α,ß-meATP, likewise, atropine inhibited CCh-induced contractions, which confirmed the effectiveness of the two inhibitors.

**Figure 3:**
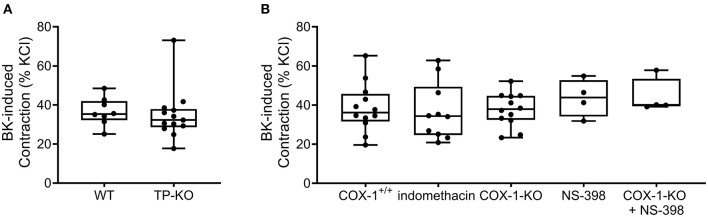
Bradykinin induces detrusor muscle contractions independently of COX-derived prostanoids. Contractile responses evoked by BK (10^−5^ M) were not altered in the bladder strips from mice deficient for TP receptors compared to those from WT mice. **(A)** The presence of the non-specific COX inhibitor indomethacin (10^−5^ M, 20 min) did not change BK-induced contractions. Furthermore, deficiency for COX-1 enzymes or treatment with the specific COX-2 inhibitor (NS-398, 10^−5^ M, 20 min) as well as their combination (COX-1-KO+NS-398) failed to influence the contractile effects elicited by BK. **(B)** (**A**: Mann-Whitey test, **B**: Kruskal-Wallis test). Case numbers: **(A)**: WT: *n* = 8/4, TP-KO: *n* = 13/4, **(B)**: COX-1^+/+^: *n* = 12/4 indomethacin: *n* = 10/5, COX-1-KO: *n* = 12/4, NS-398: *n* = 4/2, COX-1-KO + NS-398: *n* = 4/2.

Data gained from human airway smooth muscle tissues suggested that BK may constrict bronchial smooth muscle through thromboxane A_2_ release resulting in TP receptor activation (1, 35). Thus, we also performed experiments with TP-KO mouse bladders, however, their contractile responses to BK were similar to those of the control bladder strips (Figure 3A).

Next, we aimed to analyze the potential involvement of other prostanoids in mediating the effect of BK. However, incubation of the smooth muscle strips with either the nonselective COX enzyme inhibitor indomethacin (10^−5^ M, 20 min) or with the selective COX-2 inhibitor NS-398 (10^−5^ M, 20 min) failed to alter the contractile responses induced by BK (Figure 3B). For studying the function of the COX-1 enzyme selectively, COX-1-KO mouse detrusor muscle tissues were subjected to BK, however, the contractions were unaffected in the absence of COX-1 enzyme as well. As COX-1 enzyme deletion may be compensated by COX-2 enzyme upregulation (36–38), we treated COX-1-KO bladders with NS-398 to address the possibility of such compensatory mechanism. Again, we found that the contractile effect of BK was unaltered, indicating that neither COX-1 nor COX-2 appears to be involved in mediating the response (Figure 3B).

**Figure 4:**
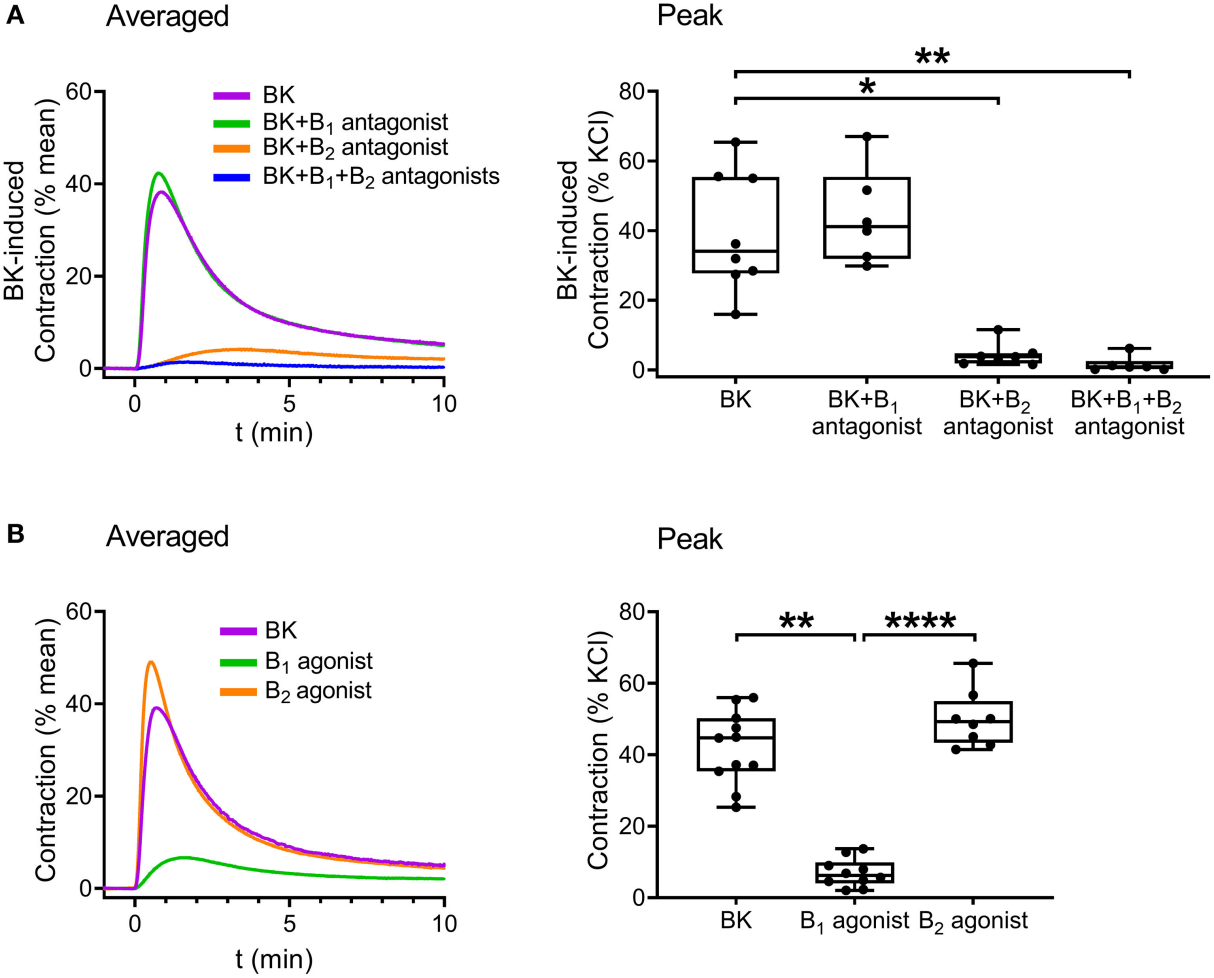
Role of B_2_ receptors in mediating BK-induced detrusor muscle contraction in murine urinary bladder strips. The BK (10^−5^ M)-induced contraction was abolished by the B_2_ receptor-specific antagonist HOE-140 (10^−6^ M, 20 min), whereas it failed to be reduced by the B_1_ receptor antagonist R-715 (10^−6^ M, 20 min). **(A)** The B_2_ receptor agonist (10^−5^ M) induced contractions of the same magnitude as BK, whereas the B_1_ receptor agonist (10^−5^ M) evoked only minor bladder contractions. **(B)** (**A,B**: Kruskal-Wallis test, *p < 0.05, **p < 0.01, ****p < 0.0001). Case numbers: **(A)**: BK: *n* = 8/8, B_1_ antagonist: *n* = 6/6, B_2_ antagonist: *n* = 7/7, B_1_ + B_2_ antagonist: *n* = 6/6, **(B)**: BK: *n* = 11/11, B_1_ agonist: *n* = 10/8, B_2_ agonist: *n* = 8/7.

Following verification of the direct contractile effect of BK in detrusor muscle, we investigated the role of B_1_ and B_2_ receptors in the contraction by applying the specific B_1_ receptor antagonist R-715 (10^−6^ M, 20 min incubation) and B_2_ receptor antagonist HOE-140 (10^−6^ M, 20 min incubation). Bradykinin-induced contractions were strongly inhibited by HOE-140, whereas R-715 failed to reduce them, implying that B_2_ receptors play the main role in mediating the effect of BK in UBSM (Figure 4A). Furthermore, simultaneous application of the two inhibitors abolished the contractile responses. Smooth muscle strips were also treated with specific agonists of B_1_ and B_2_ receptors. B_1_ agonist Lys-[Des-Arg^9^]-bradykinin (10^−5^ M) elicited only minor smooth muscle tone elevation, whereas the B_2_ agonist [Phe^8^Ψ(CH-NH)-Arg^9^]-bradykinin (10^−5^ M) had a potent constrictor effect in murine UBSM comparable to that of BK (Figure 4B) verifying the predominant role of B_2_.

**Figure 5:**
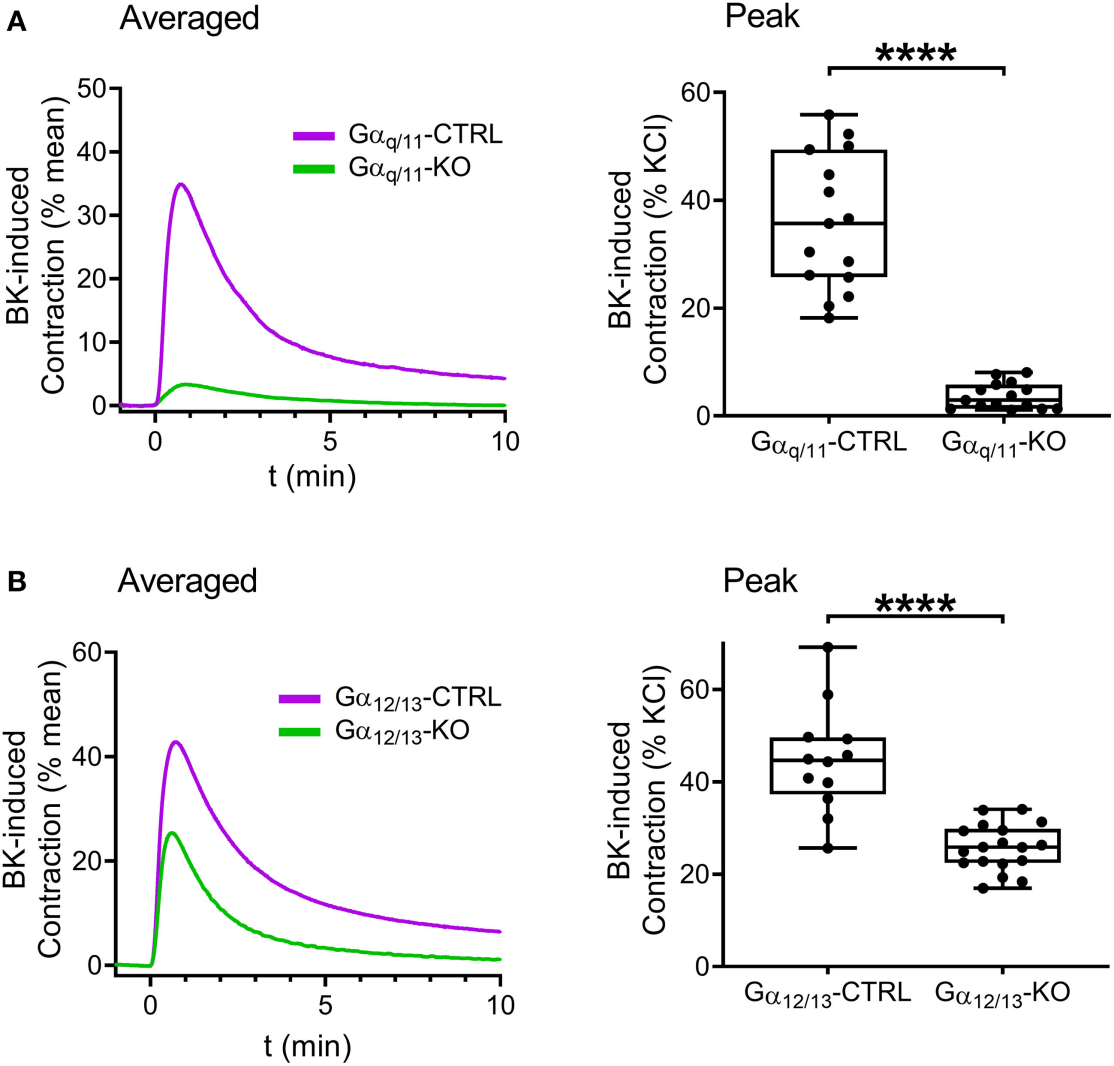
Gα_q/11_ and the Gα_12/13_ G proteins mediate the effects of BK in murine urinary bladder detrusor muscle. Contractile responses evoked by BK (10^−5^ M) were markedly reduced in the UBSM from Gα_q/11_-KO mice compared to bladder strips from Gα_q/11_-CTRL. **(A)** The detrusor contractions elicited by BK were also diminished in the UBSMs from Gα_12/13_-KO compared to the strips from Gα_12/13_-CTRL animals. **(B)** (**A,B**: Mann-Whitney test, ****p < 0.0001). Case numbers: **(A)**: Gα_q/11_-CTRL: *n* = 15/4, Gα_q/11_-KO: *n* = 15/4, **(B)**: Gα_12/13_-CTRL *n* = 12/3, Gα_12/13_-KO: *n* = 18/4.

**Figure 6:**
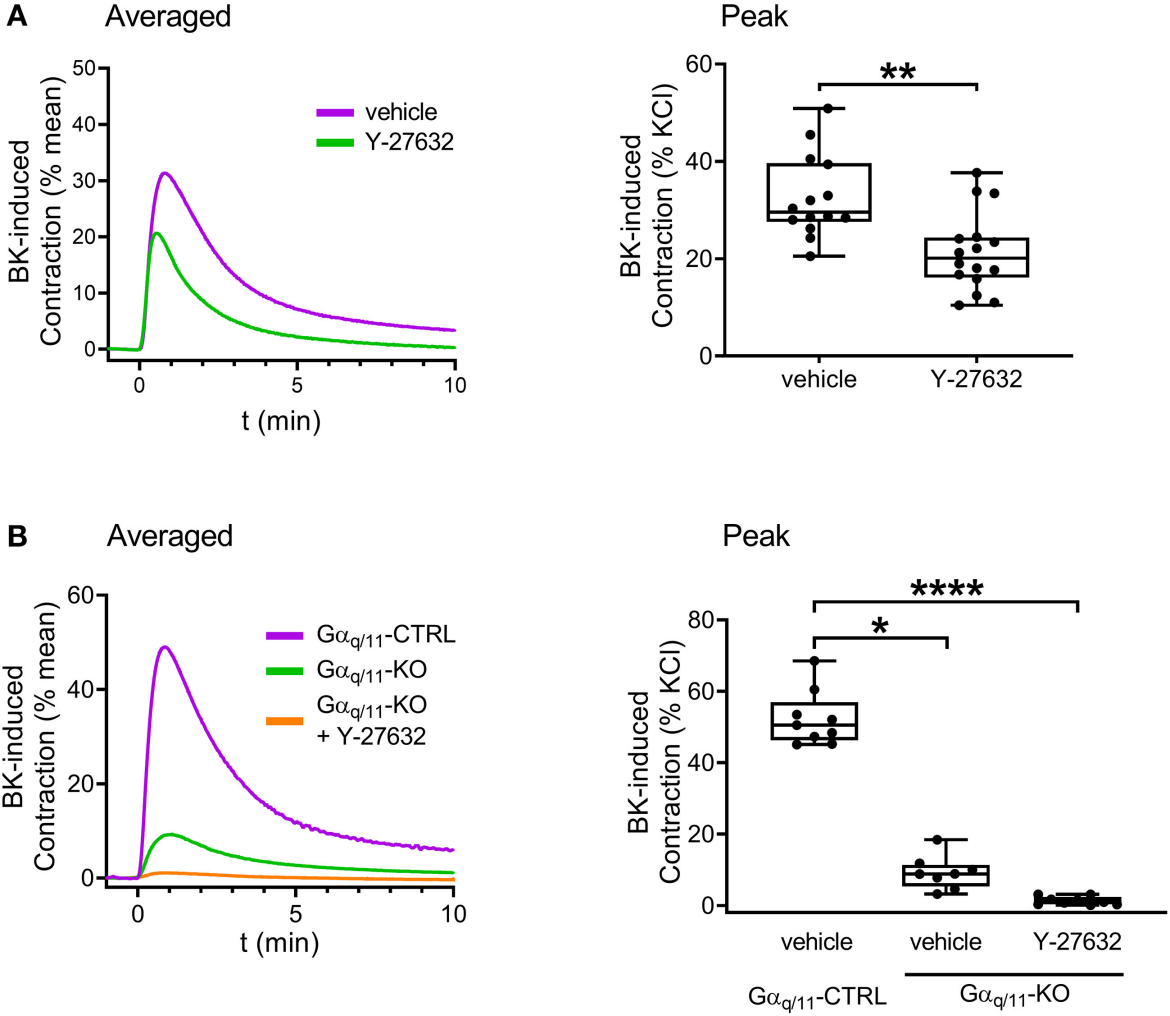
Role of Gα_12/13_-RhoA-ROCK pathway in mediating BK-induced contractions in murine UBSM. The contractile responses induced by BK (10^−5^ M) were decreased in the presence of the ROCK inhibitor (Y-27632, 10^−5^ M, 20 min). **(A)** The addition of Y-27632 (10^−5^ M, 20 min) completely suppressed the remaining BK-induced contractile responses in UBSM strips from Gα_q/11_-KO mice. **(B)** (**A**: Mann-Whitney test, **B**: Kruskal-Wallis test, *p < 0.05, **p < 0.01, and ****p < 0.0001). Case numbers: **(A)**: vehicle: *n* = 14/8, Y-27632: *n* = 16/8, **(B)**: vehicle: *n* = 9/4, Gα_q/11_-KO + vehicle: *n* = 8/4, Gα_q/11_-KO + Y-27632: *n* = 9/4.

Next, we intended to examine the contribution of Gα_q/11_- and Gα_12/13_-mediated pathways to the constriction evoked by B_2_ receptor activation. Contractile responses induced by BK (10^−5^ M) were reduced in Gα_q/11_-deficient UBSM compared to those of Gα_q/11_-CTRL murine bladders (Figure 5A). Interestingly, BK-induced contractions were also decreased in Gα_12/13_-KO mouse bladder (Figure 5B). Therefore, we aimed to investigate further the downstream signaling of Gα_12/13_-activation via application of the ROCK inhibitor Y-27632 (10^−5^ M, 20 min), which also reduced BK-evoked contractions in murine bladders (Figure 6A). In addition, the presence of Y-27632 (10^−5^ M, 20 min) completely abolished BK-induced contractions in Gα_q/11_-KO UBSM (Figure 6B).

### Signaling Pathways of Bradykinin-Induced Contractions in the Human Urinary Bladder

**Figure 7:**
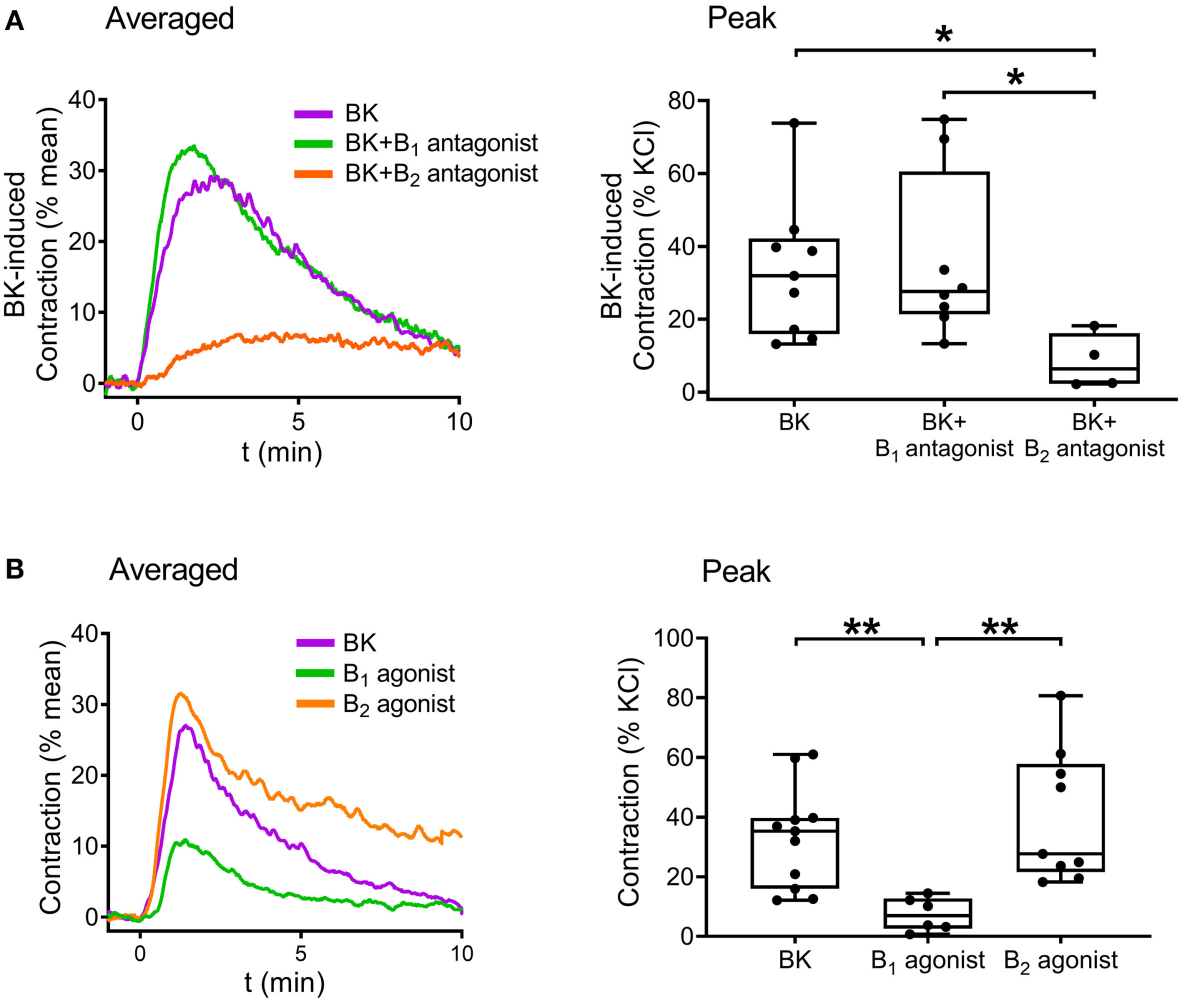
Bradykinin evokes concentration-dependent smooth muscle contraction in human urinary bladder mediated mostly by B_2_ receptors. Contractile responses evoked by BK (10^−5^ M) in human detrusor muscle were almost completely abolished in the presence of the B_2_ receptor antagonist HOE-140 (10^−6^ M, 20 min), whereas the B_1_ receptor antagonist R-715 (10^−6^ M, 20 min) failed to reduce BK-induced contractions. **(A)** The B_2_ receptor agonist (10^−5^ M) induced contractions similarly to BK, whereas the B_1_ receptor agonist (10^−5^ M) had only minor contractile activity in human detrusor muscle strips, similarly to our murine results. **(B)** (**A,B**: Kruskal-Wallis test, *p < 0.05, **p < 0.01). Case numbers: **(A)**: BK: *n* = 9/3, B_1_ antagonist: *n* = 8/3, B_2_ antagonist: *n* = 4/3, **(B)**: BK: *n* = 11/4, B_1_ agonist: *n* = 6/3, B_2_ agonist: *n* = 9/3.

**Figure 8:**
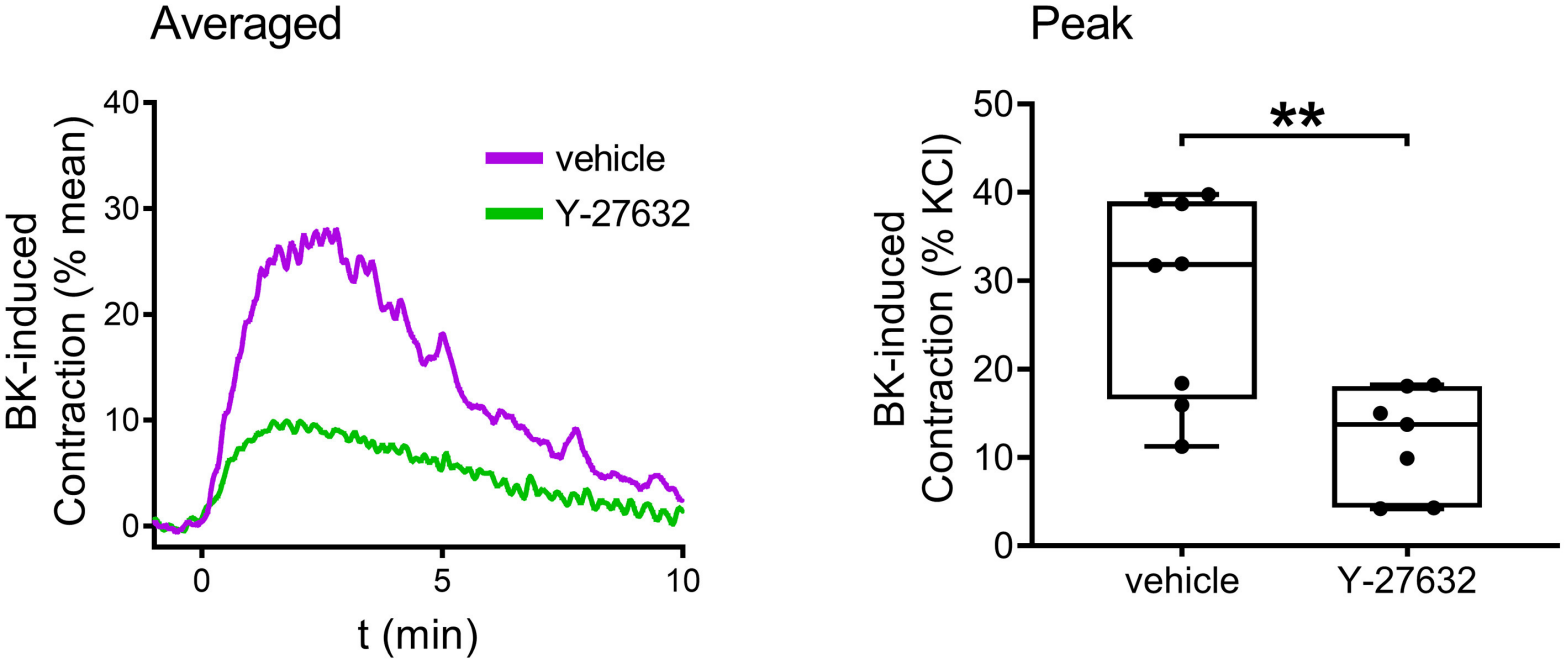
Role of the RhoA-ROCK pathway in mediating human detrusor muscle contraction evoked by BK. The ROCK inhibitor Y-27632 (10^−5^ M, 20 min) markedly reduced the contractions elicited by BK (10^−5^ M) in human UBSM, similarly to its effect in mouse bladder (Mann-Whitney test, **p < 0.01). Case numbers: vehicle: *n* = 8/2, Y-27632: *n* = 7/2.

Finally, the signaling pathways of BK in the human bladder were investigated. The contribution of BK receptor subtypes to the evoked contractions was examined by applying specific B_1_ and B_2_ receptor antagonists. The presence of the B_2_ antagonist HOE-140 (10^−6^ M, 20 min) almost completely abolished BK-induced contractions, whereas the B_1_ antagonist R-715 (10^−6^ M, 20 min) failed to alter contractions, indicating that B_2_ receptors play a prominent role in mediating BK-induced contractile responses of human UBSM as well (Figure 7A). Furthermore, the same selective B_1_ and B_2_ receptor agonists were applied to the human bladder strips as in the case of the murine experiments. The B_2_ agonist (10^−5^ M) evoked contractions approximately of the same magnitude as BK, however, the B_1_ agonist (10^−5^ M) had only a minor constricting activity compared to them (Figure 7B). Bladder strips were also treated with Y-27632 (10^−5^ M, 20 min) to examine the involvement of ROCK in mediating the effect of BK, and similarly to our murine results, the ROCK inhibitor decreased BK-induced contractile responses of the human detrusor muscle as well (Figure 8).

## Discussion

Bradykinin has been suggested as a potential mediator of disorders affecting the lower urinary tract, especially the urinary bladder (39, 40). Hence, we intended to outline the effect of BK in murine and human detrusor muscle, focusing on signal transduction of BK's actions in UBSM.

The present study demonstrates that BK evokes a concentration-dependent contraction in murine as well as in human detrusor muscle, although slight differences were observed in the contractile responses in the two species. Bradykinin contracts the mouse bladder smooth muscle with higher potency compared to that of humans (EC_50_: 1.2 × 10^−6^ M and 5.1 × 10^−6^ M, respectively), and the maximum of the contractile responses was also slightly higher in the case of mouse than in human bladders (E_max_: 52 and 42%, respectively), although these differences were not statistically significant. Though, the differing values could perhaps be attributed to interspecies variance of BK receptor expression. Another notable difference between the two species regards the characteristics of contractions elicited by the receptor agonists and the high-concentration potassium solution. The decay of contractions evoked by either CCh, BK, α,ß-meATP, or KCl in human bladder required a longer time period compared to contractions evoked in mouse detrusor strips.

Although BK has been reported to induce changes in bladder functions via activation of neuronal circuits, our results obtained by using either the muscarinic receptor antagonist atropine or the purinergic receptor antagonist PPADS indicate that neither cholinergic nor purinergic neurotransmission contributes to the contractile effect of BK in our experimental settings. Accordingly, tetrodotoxin also failed to inhibit BK-induced contractions in rabbit detrusor muscle (41). Thus, BK's contractile effect appears to be independent of releasing neurotransmitters via activating BK receptors on nerve endings.

As it has been proposed previously that BK's effects may be mediated via prostaglandin (PG)-release, we were intrigued to know whether these mediators contribute to the BK-induced contractions in murine UBSM. Treatment of the bladder strips with the non-isoform-selective COX inhibitor indomethacin failed to change contractions in response to BK. According to previous studies, BK is more prone to exert its contractile effect via COX-1 enzyme activation and resultant PG release (21). However, under our experimental conditions, BK's contractile activity remained unaltered in COX-1-KO mouse UBSM strips. Moreover, neither the presence of COX-2 inhibitor NS-398 nor simultaneously abolishing the signaling of COX-1 and COX-2 isoenzymes via the addition of NS-398 to COX-1-KO bladder strips affected BK-induced contractions. Although a role of thromboxane A_2_ has also been suggested in BK-evoked smooth muscle contraction (1, 42), our results gained from TP-KO mouse bladders indicate that TP receptors are not involved in UBSM responses induced by BK. Based on the experiments summarized above, we have concluded that neither COX-1-nor COX-2-derived prostanoids play a significant role in BK-induced contractile effects in the mouse bladder. This is rather surprising, as several studies reported that BK's smooth muscle contractile effect may be indirect and is a result of secondary PG release (21, 43). However, it has also been proposed that BK-induced prostanoid release originates from the urothelium (44), which could not be evaluated in our present study as the urothelium had been intentionally removed during the preparation of the UBSM strips.

B_2_ receptor antagonist HOE-140 diminished the contractions almost completely, whereas the presence of B_1_ antagonist R-715 left BK's effect unchanged, indicating that B_2_ receptors mediate the BK-induced contractions in the murine detrusor muscle. Our conclusion is in line with expression data implying that B_2_ is the predominant BK receptor subtype in the UBSM under physiological conditions (45). Interestingly, using the B_1_ and B_2_ inhibitors concurrently completely abolished BK-induced contractions, indicating that in the absence of B_2_ receptors, the weak contractile effect mediated by B_1_ receptors was unmasked. However, this weak contractile effect may increase significantly when B_1_ receptors are upregulated under pathological conditions (9, 19).

The predominant role of B_2_ receptors in mediating BK-evoked contractions has also been proposed by Fabiyi and Brading in whole murine bladders (17). Interestingly, the EC_50_ value calculated for BK's concentration-response curves is different in our study compared to that published by Fabiyi and Brading (1.2 × 10^−6^ and 9 × 10^−8^ M, respectively), which, perhaps, is the result of the differences in the experimental setups (isolated detrusor strips vs. whole bladders).

Verification of the B_2_ receptor-mediated effect of BK in mouse UBSM was followed by exploring the intracellular signaling involved. Our experiments with Gα_q/11_-KO or Gα_12/13_-KO mouse bladder strips proved that both Gα_q/11_ and Gα_12/13_ protein deficiency results in reduced contractile effect of BK in murine UBSM. These findings indicate that both Gα_q/11_- and Gα_12/13_-coupled signaling pathways mediate contractions induced by BK via B_2_ receptors, which appears to be a specific feature of the UBSM, as most functions of B_2_ receptors are mediated exclusively by Gα_q/11_ proteins.

It has been demonstrated that the expression of ROCK enzyme in the urinary bladder is relatively high (46), and it may contribute to elevated bladder contractions in pathological conditions as mentioned previously in the introduction, thus we also investigated the role of ROCK inhibitor Y-27632 in BK-induced mouse detrusor contractions. As the inhibitor reduced BK-evoked contractions, we concluded that the ROCK enzyme plays an important role in the intracellular signaling of BK's contractile effect in the mouse bladder. Interestingly, however, Y-27632 also inhibited contractions elicited by CCh, α,ß-meATP, and 124 mM KCl solution, whereas it did not affect the resting bladder tone (Supplementary Figure 4 at https://doi.org/10.6084/m9.figshare.16815082.v5). These results show that the signaling pathways of the contractions evoked by various stimuli share a common effector with that of BK, the ROCK enzyme. An alternative interpretation of these findings could be that Y-27632 non-selectively decreases all contractions in the urinary bladder, and therefore our experimental data does not prove the involvement of ROCK in mediating the effects of BK. However, it is noteworthy that the involvement of the Rho-ROCK pathway has also been demonstrated in BK-induced contractions of the vascular and the airway smooth muscles (47–49). On the other hand, Ribeiro et al. reported recently that inhibition of ROCK by Y-27632 failed to alter the effect of BK in the pig intravesical ureter, indicating that not all BK-induced contractile effects in the lower urinary tract are sensitive to Y-27632 (3).

Finally, we simultaneously inhibited the two G-protein mediated signaling pathways by administration of Y-27632 to Gα_q/11_-KO bladders. As BK failed to elicit contraction under these conditions, we concluded that the Ca^2+^-dependent Gα_q/11_- and the Ca^2+^-sensitizing Gα_12/13_-pathways are the exclusive mediators of BK-evoked contractions in mouse UBSM, which we believe is a major conclusion of this paper.

Our observations regarding BK's contractile effect in murine UBSM and the associated pathways established a solid base for studying BK's role in human bladder smooth muscle. Although a few studies are available reporting the contractile effect of BK in human urinary bladder (50, 51), to our knowledge, no data have been presented regarding details of BK's contractile mechanism in human UBSM.

Our findings upon application of the selective BK receptor antagonists indicate that BK evokes its contractile response predominantly via B_2_ receptors in human bladder, which is in line with our murine results. This conclusion is affirmed by data gained via BK receptor agonist administration, as the B_2_ receptor agonist evoked contractions of the same magnitude as BK, in contrast to the B_1_ receptor agonist, which had only moderate contractile activity in human detrusor muscle. The prominent role of B_2_ receptors in BK-induced human UBSM contraction is also supported by data published by Belucci et al. (11), who demonstrated that mostly B_2_ receptors are expressed in human cultured detrusor smooth muscle cells. Nevertheless, one should note that our findings rely on experiments performed on tumor-free, healthy bladder tissues without exhibiting any signs of obvious pathological alterations. As mentioned previously, while B_2_ receptors are expressed constitutively in healthy tissues, B_1_ receptors' expression increases rather as a response to tissue injury or inflammation. Thus, the ratio of the two BK receptor's contribution to detrusor contraction evoked by BK may be altered under pathological conditions with increased B_1_ receptor expression in UBSM.

Based on our results in murine bladder, we predicted the role of ROCK within the intracellular signaling of B_2_ receptor-mediated detrusor muscle contraction also in humans. Indeed, our experiments with the ROCK-inhibitor Y-27632 demonstrate that ROCK plays a major role in BK-evoked contraction in human UBSM, as the inhibitor decreased contractile responses induced by BK. This is in accordance with our corresponding murine experiments, furthermore, we believe that this is the first study demonstrating that ROCK is a key mediator of BK's contractile activity in human UBSM. Similar to mouse experiments, the ROCK inhibitor reduced contractions induced by CCh, α,ß-meATP, and 124 mM KCl as well (Supplementary Figure 5 at https://doi.org/10.6084/m9.figshare.16815088.v5).

Taken together, this report presents for the first time a thorough investigation of signaling pathways contributing to BK's contractile effect in mouse and human detrusor muscle. It has been clarified that this effect results from BK acting predominantly on UBSM B_2_ receptors. The contractions are independent of cholinergic or purinergic neurotransmission as well as prostanoids. The role of the simultaneous contribution of G_q/11_ and G_12/13_ protein-coupled pathways to the contraction was also demonstrated. Furthermore, the ROCK enzyme within the G_12/13_ protein signaling pathway also proved to be an important mediator, as its inhibition markedly decreased BK-induced contractions of both the murine and the human bladder. Interestingly, inhibition of ROCK has been shown to increase bladder compliance under both resting and stretched conditions, and ROCK inhibitors induce a relaxation of the bladder smooth muscle (52). Therefore, ROCK inhibitors represent a promising new tool for the treatment of detrusor overactivity. However, the appropriate dosage would definitely be an important issue and potentially a limitation of the use of ROCK inhibitors for the treatment of voiding disorders, as the normal micturition also appears to involve ROCK activation.

In conclusion, research on the role of the B_2_ receptor as well as ROCK in UBSM contraction may become a promising field for further studies and contribute to a better understanding of urinary bladder physiology and pathophysiology, which may lead to the development of novel drugs for the treatment of voiding disorders.

## Data Availability Statement

The raw data supporting the conclusions of this article will be made available by the authors, without undue reservation.

## Ethics Statement

The studies involving human participants were reviewed and approved by Scientific and Research Committee of the Medical Research Council of Hungary. The patients/participants provided their written informed consent to participate in this study. The animal study was reviewed and approved by Government Office of Pest County.

## Author Contributions

KB, HB, PJM, PN, SO, and ZB conceived and designed research. AH and AK performed radical cystectomies and helped in patient selection. IK performed the pathological examination of the human urinary bladder samples. KB, HB, PJM, ÁL, and ZB performed experiments. KB and HB analyzed data, prepared figures, and drafted manuscript. KB, HB, PJM, IK, AH, AK, AM, PN, SO, and ZB interpreted the results of experiments. KB, HB, PJM, AM, PN, SO, and ZB edited and revised manuscript. KB, HB, PJM, ÁL, IK, AH, AK, MR, AM, PN, SO, and ZB approved the final version of manuscript.

## Funding

This research was funded by the Hungarian National Research, Development and Innovation Office (K-125174, K-135683, K-139230, 2020-1.1.6-JÖVÖ-2021-00010, TKP2021-EGA-25, and NVKP_16-1-2016-0042 grants) as well as by the Higher Education Institutional Excellence Program of the Ministry of Human Capacities in Hungary, within the framework of the Molecular Biology thematic program of the Semmelweis University, and supported by the EFOP-3.6.3-VEKOP-16-2017-00009 and ÚNKP-19-2-I-SE-48 grants as well as by Gedeon Richter Plc Talent Foundation. The Central Library of Semmelweis University also kindly provided fund for open access publication fees.

## Conflict of Interest

The authors declare that the research was conducted in the absence of any commercial or financial relationships that could be construed as a potential conflict of interest.

## Publisher's Note

All claims expressed in this article are solely those of the authors and do not necessarily represent those of their affiliated organizations, or those of the publisher, the editors and the reviewers. Any product that may be evaluated in this article, or claim that may be made by its manufacturer, is not guaranteed or endorsed by the publisher.

## Abbreviations

α, β-meATP, α: β-methyleneadenosine 5′-triphosphate
ACh: acetylcholine
ATP: adenosine triphosphate
BK: bradykinin
CCh: carbachol
COX: cyclooxygenase
Cre-ERT2: cre-recombinase with a modified estrogen receptor-binding domain
CTRL: control
DAG: diacylglycerol
DMSO: dimethyl sulfoxide
GPCR: G protein-coupled receptor
HBSS: Hank's Balanced Salt Solution
KO: knockout
MLC: myosin light chain
OAB: overactive bladder
PG: prostaglandin
PLC-β: phospholipase C-β
PPADS, pyridoxalphosphate-6-azophenyl-2′: 4′-disulfonate
ROCK: Rho-kinase
SMMHC: smooth muscle myosin heavy chain
SR: sarcoplasmic reticulum
TP: thromboxane prostanoid
UBSM: urinary bladder smooth muscle
WT: wild-type.
